# Diabetes Type 2: Circulating Phosphatidylserine-Expressing Platelets Regulate Whole Blood Agonist-Evoked Platelet Activity In Vitro

**DOI:** 10.1055/a-2900-2080

**Published:** 2026-07-21

**Authors:** Petter Järemo, Magnus Oweling, Maria Edvardsson

**Affiliations:** 1 Jaremo Vardbolag ABNorrköpingSweden; 2 NärsjukvårdenLocal Healthcare CentreFinspångSweden; 3 Primary Health Care CentreDepartment of Clinical ChemistryLinköping UniversityLinköpingSweden; 4 Primary Health Care CentreDepartment of Biomedical and Clinical SciencesLinköping UniversityLinköpingSweden

**Keywords:** ADP, thrombin, cross-linked collagen-related peptide, diabetes type 2, fibrinogen receptor, flow cytometry, lysosomal exocytosis, phosphatidylserine, platelets, platelet reactivity

## Abstract

**Background** Platelet agonists responses in vitro (i.e., reactivity) include the creation of phosphatidylserine (PS)-exposing platelets together with the activation of the fibrinogen receptors (α _IIb_ β _3_ ) and lysosomal exocytosis. It is feasible to judge the activation pathways by analyzing platelet surface annexin V, the activated fibrinogen receptor (PAC-1), and the release of lysosomal-associated membrane protein (LAMP-1), correspondingly. We postulate that, in type 2 diabetes (T2DM), surface PS of circulating platelets, unprovoked in vitro *,* links with whole blood (WB) agonist-induced responses.

**Patients and Methods** After informed consent, T2DM subjects ( *n* = 35) were enrolled. A Percoll gradient (1.09–1.04 kg/L) separated their normal-sized platelets according to density into subpopulations ( *n* = 8). A flow cytometer analyzed surface annexin V (mean fluorescence intensity [MFI]) of the subfractions, unprovoked ex vivo. The datasets were subsequently correlated with platelet WB agonist-induced responses (i.e., annexin V, PAC-1, and LAMP-1 [all MFI]) to α-thrombin (10 μM), cross-linked collagen-related peptide (CRP-XL, 0.15 μg/mL), and adenosine diphosphate (ADP, 5 μM) in vitro.

**Results** Surface annexin V (MFI) of most platelet subfractions and the magnitudes of WB agonist-induced annexin V (MFI) of normal-sized platelets in vitro associated closely. Such PS-expressing platelets also linked inversely with WB agonist-evoked surface PAC-1 (MFI) (all used agonists) and LAMP-1 (MFI) (CRP-XL, ADP only). It is concluded that surface PS, unprovoked in vitro, of most density-separated platelets connected with platelet reactivity, i.e., their WB agonist-evoked reactions in the test tube.

## Introduction

It is strongly agreed that platelets exhibit different behaviors in response to agonists. Some demonstrate an activation-induced redistribution of phosphatidylserine that promotes the assembly of platelet membrane prothrombinase complexes and thrombin generation. 1 2 3 4 Further, aggregatory platelet subpopulations stabilize the primary platelet plug by bridging fibrinogen to their activated receptors (αIIbβ3). 5 6 Finally, other platelets liberate granule stored adhesion molecules. Examples include release of α-granules and inorganic polyphosphate. 7 8 9 Key regulators of platelet responses in vitro (i.e., reactivity) remain unknown.

Recently, there has been a renewed awareness of platelet variability, prompting a meticulous examination of density-separated platelets. 10 11 12 The density of platelets spans the interval of 1.09 to 1.04 kg/L 10 with low-density platelets containing fewer mitochondria, granules, and glycogen. 13 14 15 Based on surface activity markers, both high- and low-density platelets circulate in a manner that is “more activated.” 12 16 Alterations in platelet density are characteristic of numerous human diseases, including Alzheimer's dementia, inflammatory bowel disease, and preeclampsia. 16 17 18 Consequently, platelet density heterogeneity influences human pathophysiology.

Platelets of type 2 diabetes (T2DM) individuals exhibit increased density and larger volume with size increasing well before diabetic complications develop. 19 20 In response to agonists, platelets from diabetic patients demonstrate accelerated aggregation. 21 22 Improved HbA1c normalizes both mean platelet volume and reactivity and provides a plausible link between metabolic control and T2DM complications. 22 23 24 The regulation of T2DM platelet responses to agonists in the test tube remains unclear. This study hypothesized that circulating phosphatidylserine-expressing platelets unstimulated in vitro influence whole blood (WB) agonist-provoked reactions, i.e., platelet surface phosphatidylserine, fibrinogen receptor activity, and lysosomal exocytosis, i.e., reactivity.

## Methods

### Patients and Whole Blood Collection

After informed consent in writing T2DM individuals ( *n* = 35) were included. The T2DM diagnosis was set by the primary care physician. Key demographic and clinical variables are given in Table 1 . The participants had appointments with their family physicians and entered the study, if laboratory personnel (the coauthor M.E.) was available. No exclusion criteria were imposed. EDTA-anticoagulated WB (3 mL) was drawn for routine biochemistry ( Table 1 ). For flow cytometry studies and platelet density separations, WB (each 9 mL) was sampled from the antecubital vein into sodium citrate test tubes (3.2%). The flow cytometry work began approximately 2 h after WB collection.

**Table 1:** Baseline characteristics of the type 2 diabetes subjects ( *n* = 35).

	Diabetes type 2 patients
Age (y)	72 ± 11 (SD)
Body weight (kg)	83 ± 13 (SD)
Female/male ( *n* )	20/15
Systolic blood pressure (mm Hg)	129 ± 11 (SD)
Previous cerebral disease ( *n* )	1
Previous coronary disease ( *n* )	2
Insulin ( *n* )	11
Metformin ( *n* )	19
Other oral antidiabetics ( *n* )	11
Angiotensin-converting enzyme inhibitors ( *n* )	12
Angiotensin type 2 receptor inhibitors ( *n* )	6
Aspirin ( *n* )	8
β-blockers ( *n* )	10
Ca ^2+^ -blockers ( *n* )	10
Clopidogrel ( *n* )	1
Diuretics ( *n* )	12
Statins ( *n* )	22
Creatinine (μmol/L)	81 ± 27 (SD)
Hemoglobin A1 _c_ (mmol/L)	58 ± 12 (SD)

Abbreviation: SD, standard deviation.

Note: Recent prescriptions of ( *n* = 5) subjects were lacking.

### Platelet Density Separation

Citrate-anticoagulated WB was used to separate platelets based on their density. It—9 parts—was combined with a mixture (1 part) that included equal amounts of these stock solutions. 10 18

- Prostaglandin E _1_ (1 mg/L) dissolved in 95% (w/v) ethanol.
- Na _2_ EDTA (0.13M) and Na _2_ Citrate (0.15M) (pH 7.4 at 25 °C).
- Theophylline (2.7 mM) dissolved in TRIS chloride buffer (150 mM) (pH 7.4 at 25 °C).

Percoll, i.e., linear polyvinylpyrrolidone-coated silica gradients with the density span 1.04–1.09 kg/L, were used to separate platelets according to density. 10 16 18 After centrifugation, the gradients were split into subfractions ( *n* = 8). 12 In this setting, low-digit samples denote high-density platelets.

### Flow Cytometry

We used a multicolor Gallio flow cytometer (Beckman Coulter, Brea, California, United States) equipped with lasers ( *n* = 3) (638, 488, 405 nm). Mean fluorescence intensity (MFI) and the proportion (%) (ratio) of fluorescence-positive platelets served as experimental parameters. The following surface features (both MFI, ratio) of normal-sized platelets were investigated, and throughout the study and for each activation parameter (MFI and ratio), data were treated separately.

- Surface annexin V assessed phosphatidylserine (both on subfractions and in WB).
- Platelet PAC-1 activity judged the activated fibrinogen receptor (αIIbβ3) (WB only).
- LAMP-1 expressions estimated lysosomal exocytosis (WB only).

A previously described flow cytometry protocol was employed. 1 11 12 In this study, technical issues with a new antibody prevented us from measuring platelet P-selectin expression. Table 2 displays comprehensive data about antibodies and probes. When determining platelet reactivity in the test tube α-thrombin (10 μM), cross-linked collagen-related peptide (CRP-XL) (0.15 μg/mL), and ADP (5 μM) served as agonists. After 10 min, the reactions were terminated with HEPES-Ca. 2 The flow cytometer gating has been described previously. 1 11 12 Glycoprotein IIb receptor fluorescence and particle size identified normal-sized platelets. When analyzing subfraction platelets (annexin [MFI, ratio]), particle acquisition finished either after 2 min or when corpuscle counts exceeded 5000. Thus, the evaluated platelets varied according to subfraction platelet counts.

**Table 2:** Flow cytometry reagents, i.e., antibodies, probes, agonists, final concentrations, and manufacturers of the reagents.

Antibody	CD41; phycoerythrin (0.69 μg/mL)
Platelet identification	
Detection of the platelet receptor α _IIb_	
Beckman Coulter (Brea, California, United States)	
Probe	Annexin V-V450; phycoerythrin (2.67 ng/mL)
Surface attached annexin V	
Analysis of membrane-exposed phosphatidylserine	
Becton Dickinson (Franklin Lakes, New Jersey, United States)	
Antibody	PAC-1; fluorescein isothiocyanate (0.56 μg/mL)
Fibrinogen α _IIb_ β _3_ receptor activity	
Analysis of membrane-bound PAC-1	
Beckman Coulter (Brea, California, United States)	
Antibody	CD107a, clone: H4A39; phycoerythrin (0.5 μg/mL)
Lysosomal exocytosis	
Analysis of membrane-bound lysosomal-associated membrane protein (LAMP-1)	
Becton Dickinson (Franklin Lakes, New Jersey, United States)	
Agonist	(10 μM)
α-thrombin	
Proteinase-activated receptor 1	
Sigma-Aldrich (St. Louis, Michigan, United States)	
Agonist	(0.15 μg/mL)
Cross-linked collagen-related peptide (CRP-XL)	
University of Cambridge (Cambridge, UK)	
Agonist	(5 μM)
Adenosine diphosphate (ADP)	
Sigma-Aldrich (St. Louis, Michigan, United States)	

### Evaluation of Experimental Data

Surface phosphatidylserine as judged from annexin V (MFI, ratio) of circulating normal-sized density separated platelet fractions ( *n* = 8), nonstimulated ex vivo, was determined. The study then compared subfraction annexin V (both MFI, ratio) and WB normal-sized agonist-evoked (α-thrombin [10 μM], CRP-XL [0.15 μg/mL], ADP [5 μM]) platelet activation as estimated from the following activation parameters: surface annexin V, PAC-1, and lysosomal-associated membrane protein-1 (LAMP-1) (all MFI, ratio). The correlation coefficient was employed to compare subfraction annexin V (both MFI, ratio) and WB agonist-evoked activity markers (both MFI, ratio). In addition, for every WB agonist-evoked activity marker (both MFI, ratio), T2DM participants ( *n* = 35) were divided into groups (reactivity _high_ and _low_ ) consisting of *n* = 17 and *n* = 18 subjects, respectively. Tables 3 and 4 provide for each agonist-provoked surface activity marker for normal-sized platelets numeric values the reactivity _high_ and _low_ groups. The two groups were compared with respect to subfraction surface annexin V (both MFI, ratio). Consequently, both the correlation coefficient and the unpaired Student’s *t* test were utilized in the evaluation process.

**Table 3:** Whole blood agonist-evoked surface activity markers (means ± standard deviation) for the _high_ and _low_ reactivity groups (annexin V, PAC-1, LAMP-1 [left mean fluorescence intensity, right ratio]) of normal-sized platelets.

	Mean fluorescence intensity after provocation			Percentage positive cells after provocation		
	α-Thrombin	CRP-XL	ADP	α-Thrombin	CRP-XL	ADP
	(10 μM)	(0.15 μg/mL)	(5 μM)	(10 μM)	(0.15 μg/mL)	(5 μM)
Whole blood agonist-evoked annexin V of normal-sized platelets						
Reactivity _high_	0.14 ± 0.04 (SD)	0.17 ± 0.04 (SD)	0.19 ± 0.03 (SD)	3.6 ± 2.2 (SD)	7.9 ± 1.3 (SD)	4.0 ± 1.9 (SD)
Reactivity _low_	0.10 ± 0.00 (SD)	0.10 ± 0.00 (SD)	0.11 ± 0.02 (SD)	0.9 ± 0.4 (SD)	1.3 ± 0.6 (SD)	0.5 ± 0.1 (SD)
Whole blood agonist-evoked PAC-1 of normal-sized platelets						
Reactivity _high_	1.87 ± 0.11 (SD)	2.2 ± 1.3 (SD)	2.20 ± 1.3 (SD)	70 ± 25 (SD)	75 ± 17 (SD)	75 ± 19 (SD)
Reactivity _low_	0.49 ± 0.06 (SD)	0.58 ± 0.14 (SD)	0.54 ± 0.08 (SD)	18 ± 7 (SD)	30 ± 15 (SD)	25 ± 10 (SD)
Whole blood agonist-evoked LAMP-1 of normal-sized platelets						
Reactivity _high_	0.86 ± 0.09 (SD)	1.06 ± 0.16 (SD)	0.66 ± 0.11 (SD)	70 ± 9 (SD)	75 ± 8 (SD)	55 ± 9 (SD)
Reactivity _low_	0.57 ± 0.14 (SD)	0.58 ± 0.22 (SD)	0.42 ± 0.06 (SD)	39 ± 15 (SD)	39 ± 22 (SD)	24 ± 9 (SD)

Abbreviations: ADP, Adenosine diphosphate; CRP-XL, cross-linked collagen-related peptide; LAMP-1, lysosomal-associated membrane protein-1; SD, standard deviation.

**Table 4:** Density intervals and surface annexin V (mean fluorescence intensity, ratio), activity unprovoked in vitro (means ± standard deviation), for the eight normal-sized platelet density subfractions.

Fraction	Density intervals (kg/L)	Annexin V (MFI)	Annexin V (ratio)
1	1.090–1.084	0.24 ± 0.11	15.1 ± 12.5
2	1.0841–1.078	0.20 ± 0.06	6.6 ± 5.0
3	1.0781–1.071	0.20 ± 0.06	3.9 ± 2.9
4	1.0711–1.065	0.19 ± 0,05	2.9 ± 2.2
5	1.0651–1.059	0.19 ± 0.05	2.2 ± 1.7
6	1.0591–1.053	0.19 ± 0.05	3.6 ± 5.8
7	1.0531–1.047	0.22 ± 0.10	6.9 ± 8.1
8	1.0471–1.040	0.19 ± 0.06	5,8 ± 6.0

Abbreviations: CRP-XL, collagen-related peptide; surface annexin V, platelet phosphatidylserine positivity; MFI, mean fluorescence intensity; ratio, percentage positive platelets.

### Ethical Permission

The study complied with the Declaration of Helsinki and was approved by the Ethics Committee of the Faculty of Health Science, Linköping University, Sweden (registration number: 2018/53.31).

## Results

Table 5 presents the associations between surface annexin V (MFI, ratio) of density-separated platelets and agonist-induced WB platelet surface annexin V ([MFI] left, [ratio] right). All *p* -values reported are independent of the correlation coefficients and pertain to comparisons of subpopulation annexin V (MFI, ratio) of reactivity _high_ and _low_ groups for each agonist-induced WB surface annexin V (MFI, ratio) of normal-sized platelets. As shown in Table 5 (left), there were statistically significant relationships between unprovoked annexin V (MFI) in vitro of density subpopulations numbers 2 to 6 and 8, and WB agonist-stimulated (α-thrombin [10 μM]: *r* range 0.5–0.6; CRP-XL [0.15 μg/mL]: *r* range 0.5–0.7; ADP [5 μM]: *r* range 0.7–0.8) platelet surface annexin V (MFI) (all *p* < 0.001).

**Table 5:** ( **A** and **B** ) Associations between baseline surface annexin V expression in normal-sized platelet subpopulations separated by density ( *n* = 8)—expressed as mean fluorescence intensity in ( **A** ) and ratio in ( **B** )—and whole blood annexin V responses induced by agonist stimulation, reported as mean fluorescence intensity (left panels) and ratio (right panels), after exposure to α-thrombin (10 μM), collagen-related peptide (0.15 μg/mL), or adenosine diphosphate (5 μM). The associations were evaluated in two ways. The correlation coefficients ( *r* ) were determined between unstimulated in vitro annexin V expression of the platelet subfractions and agonist-induced whole blood annexin V responses of normal-sized platelets. Second, the 35 participants were separated into high-reactivity ( *n* = 17) and low-reactivity ( *n* = 18) groups according to whole blood agonist-induced annexin V expression (mean fluorescence intensity and ratio) of normal-sized platelets. For each subfraction annexin V and each whole blood response measure, the two reactivity groups were compared using the Student’s *t* test, and the corresponding *p* -values are presented in the tables. They are independent of, and were not calculated from, the correlation analyses.

Density fractions	Mean fluorescence intensity after provocation			Percentage positive cells after provocation		
	α-Thrombin	CRP-XL	ADP	α-Thrombin	CRP-XL	ADP
	(10 μM)	(0.15 μg/mL)	(5 μM)	(10 μM)	(0.15 μg/mL)	(5 μM)
**A. Annexin V (mean fluorescence intensity) of normal-sized density-separated circulating platelets vs. whole blood agonist-evoked annexin V of normal-sized platelets**						
1	All agonists (NS)			All agonists (NS)		
2	( *r* = 0.5) ( *p* < 0.001)	( *r* = 0.6) ( *p* < 0.001)	( *r* = 0.8) ( *p* < 0.001)	All agonists (NS)		
3	( *r* = 0.6) ( *p* < 0.001)	( *r* = 0.7) ( *p* < 0.001)	( *r* = 0.8) ( *p* < 0.001)	All agonists (NS)		
4	( *r* = 0.5) ( *p* < 0.001)	( *r* = 0.6) ( *p* < 0.001)	( *r* = 0.8) ( *p* < 0.001)	All agonists (NS)		
5	( *r* = 0.6) ( *p* < 0.001)	( *r* = 0.7) ( *p* < 0.001)	( *r* = 0.8) ( *p* < 0.001)	All agonists (NS)		
6	( *r* = 0.5) ( *p* < 0.001)	( *r* = 0.6) ( *p* < 0.001)	( *r* = 0.8) ( *p* < 0.001)	All agonists (NS)		
7	All agonists (NS)			All agonists (NS)		
8	( *r* = 0.5) ( *p* < 0.001)	( *r* = 0.5) ( *p* < 0.001)	( *r* = 0.7) ( *p* < 0.001)	All agonists (NS)		
**B. Annexin V (ratio) of normal-sized density-separated circulating platelets vs. whole blood agonist-evoked annexin V of normal-sized platelets**						
1	( *r* = −0.4) ( *p* < 0.05)	(NS)	( *r =* −0.4) ( *p* < 0.05)	All agonists (NS)		
2	All agonists (NS)			All agonists (NS)		
3	(NS)	( *r* = 0.5) ( *p* < 0.05)	( *r* = 0.6) ( *p* < 0.05)	( *r* = 0.3) ( *p* < 0.05)	( *r* = 0.4) ( *p* < 0.05)	( *r* = 0.6) ( *p* < 0.05)
4	( *r* = 0.4) ( *p* < 0.05)	( *r* = 0.5) ( *p* < 0.05)	( *r* = 0.6) ( *p* < 0.05)	( *r* = 0.4) ( *p* < 0.01)	(NS)	(NS)
5	( *r* = 0.6) ( *p* < 0.001)	( *r* = 0.6) ( *p* < 0.01)	( *r* = 0.7) ( *p* < 0.01)	( *r* = 0.3) ( *p* < 0.01)	(NS)	(NS)
6–8		All agonists (NS)		All agonists (NS)		

Abbreviations: ADP, adenosine diphosphate; annexin V, platelet phosphatidylserine positivity; CRP-XL, collagen-related peptide; NS, not significant; ratio, percentage positive platelets.

Table 5 reveals that annexin V (ratio) of fraction number 1, i.e., very dense platelets, was inversely linked with WB annexin V (MFI) following α-thrombin and ADP (both *r* = −0.4) provocation. The *p* -values when comparing the subfraction activity markers of the reactivity _high_ and _low_ groups were both *p* < 0.05. Furthermore, annexin V (ratio) of semidense platelets (fractions 4, 5) was associated with WB α-thrombin-evoked annexin V (MFI) ( *r* range [0.4–0.6]). The corresponding *p* -values when comparing subfraction activity markers of the reactivity _high_ and _low_ groups were *p* < 0.05 and 0.001, respectively ( Table 5 ). The figures when comparing platelet subpopulation annexin V (ratio) and α-thrombin-provoked annexin V (ratio) were *r* range (0.3–0.4) and the *p* -values proved to be fraction number 3 ( *p <* 0.05) and numbers 5, 6 ( *p <* 0.01). Table 5 also shows that CRP-XL and ADP behaved slightly differently in that annexin V (ratio) of semidense fractions were related to WB agonist-evoked annexin V ( *r* range [0.4–0.7]) (number 3 [MFI, ratio] [ *p <* 0.05], number 4 [MFI only] [ *p <* 0.05], number 5 [MFI only] [ *p <* 0.01]).

Table 6 presents the relationships between annexin V expressions (MFI, ratio) of density-separated platelets and WB platelet surface PAC-1 (MFI [left], ratio [right]) following agonist provocation. The *p* -values related to the comparison of the PAC-1 reactivity _high_ and _low_ groups with respect to subfraction surface annexin V (MFI [left], ratio [right]). With few exceptions (fractions number 1 α-thrombin, ADP [both MFI, ratio], CRP-XL [MFI] and number 7 [both MFI, ratio] all agonists), surface annexin V (MFI) of the density fractions and agonist-evoked WB PAC-1 (both MFI, ratio) showed highly significant inverse relationships ( Table 6 ). For α-thrombin (both MFI, ratio), the *p* -values indicated less significance ( *r* range [−0.4 to −0.6] ( *p* range [ *p <* 0.05 to *p <* 0.01]; Table 6 ). For PAC-1 (MFI) reactions, the significances after CRP-XL and ADP provocations proved to be *r* = −0.6 ( *p <* 0.001). CRP-XL-provoked PAC-1 (ratio) and subfraction annexin V (MFI) displayed similar inverse associations (fractions number 1 [ *r* = −0.3] [ *p <* 0.05] and numbers 2–6, 8 [ *r* = −0.6] [ *p <* 0.001]). The comparison of subfraction surface annexin V (MFI, ratio) and ADP-evoked PAC-1 (ratio) responses yielded higher *p* -values (all *r* = −0.7) ( *p <* 0.01). For α-thrombin (both MFI, ratio), the relationships were *r* range [−0.4 to −0.6] ( *p* range [ *p <* 0.05 to *p <* 0.01]; Table 6 ).

**Table 6:** ( **A** and **B** ) Relationships between basal surface annexin V expression in normal-sized, density-fractionated platelet subpopulations ( *n* = 8)—reported as mean fluorescence intensity in ( **A** ) and as percentage positivity (ratio) in ( **B** )—and whole blood agonist-induced PAC-1 responses, expressed as mean fluorescence intensity (left panels) and ratio (right panels), following stimulation with α-thrombin (10 μM), collagen-related peptide (0.15 μg/mL), and adenosine diphosphate (5 μM). These relationships were examined in two ways. Correlation coefficients ( *r* ) were calculated between unstimulated in vitro annexin V expression in the platelet subfractions and agonist-stimulated whole blood PAC-1 responses of normal-sized platelets. Second, the 35 participants were divided into higher ( *n* = 17) and lower-reactivity ( *n* = 18) groups based on whole blood agonist-induced PAC-1 expression (both mean fluorescence intensity, ratio). For each subfraction surface annexin V of the high- and low-reactivity groups were compared using the Student’s *t* test, and the resulting *p* -values are shown in the tables. They are separate from, and not derived from, the correlation analyses.

Density fractions	Mean fluorescence intensity after provocation			Percentage positive cells after provocation		
	α-Thrombin	CRP-XL	ADP	α-Thrombin	CRP-XL	ADP
	(10 μM)	(0.15 μg/mL)	(5 μM)	(10 μM)	(0.15 μg/mL)	(5 μM)
**A. Annexin V (mean fluorescence intensity) of normal-sized density-separated circulating platelets vs. whole blood agonist-evoked PAC-1 of normal-sized platelets**						
1	All agonists (NS)			(NS)	( *r* = −0.3) ( *p* < 0.05)	(NS)
2	( *r* = −0.6) ( *p* < 0.05)	( *r* = −0.6) ( *p* < 0.001)	( *r* = −0.6) ( *p* < 0.001)	( *r* = −0.7) ( *p* < 0.05)	( *r* = −0.6) ( *p* < 0.001)	( *r* = −0.7) ( *p* < 0.01)
3	( *r* = −0.6) ( *p* < 0.01)	( *r* = −0.6) ( *p* < 0.001)	( *r* = −0.6) ( *p* < 0.001)	( *r* = −0.7) ( *p* < 0.01)	( *r* = −0.6) ( *p* < 0.001)	( *r* = −0.7) ( *p* < 0.01)
4	( *r* = −0.6) ( *p* < 0.05)	( *r* = −0.6) ( *p* < 0.001)	( *r* = −0.6) ( *p* < 0.001)	( *r* = −0.7) ( *p* < 0.05)	( *r* = −0.6) ( *p* < 0.001)	(r = −0.7) ( *p* < 0.01)
5	( *r* = −0.6) ( *p* < 0.01)	( *r* = −0.6) ( *p* < 0.001)	( *r* = −0.6) ( *p* < 0.001)	( *r* = −0.7) ( *p* < 0.01)	( *r* = −0.6) ( *p* < 0.001)	( *r* = −0.7) ( *p* < 0.01)
6	( *r* = −0.6) ( *p* < 0.01)	( *r* = −0.6) ( *p* < 0.001)	( *r* = −0.6) ( *p* < 0.001)	( *r* = −0.7) ( *p* < 0.01)	( *r* = −0.6) ( *p* < 0.001)	( *r* = −0.7) ( *p* < 0.01)
7	All agonists (NS)			All agonists (NS)		
8	( *r* = −0.6) ( *p* < 0.05)	( *r* = −0.6) ( *p* < 0.001)	( *r* = −0.6) ( *p* < 0.001)	( *r* = −0.7) ( *p* < 0.05)	( *r* = −0.6) ( *p* < 0.001)	( *r* = −0.7) ( *p* < 0.01)
**B. Annexin V (ratio) of normal-sized density-separated circulating platelets vs. whole blood agonist-evoked PAC-1 of normal-sized platelets**						
1	( *r* = 0.2) ( *p* < 0.05)	(NS)	( *r* = 0.2) ( *p* < 0.05)	( *r* = 0.5) ( *p* < 0.05)	(NS)	( *r* = 0.4) ( *p* < 0.05)
2	All agonists (NS)				All agonists (NS)	
3	(NS)	( *r* = −0.3) ( *p* < 0.05)	( *r* = −0.3) ( *p* < 0.05)	(NS)	( *r* = −0.4) ( *p* < 0.05)	( *r* = −0.4) ( *p* < 0.05)
4	( *r* = −0.2) ( *p* < 0.05)	( *r* = −0.3) ( *p* < 0.05)	( *r* = −0.3) ( *p* < 0.05)	( *r* = −0.3) ( *p* < 0.05)	( *r* = −0.4) ( *p* < 0.05)	( *r* = −0.4) ( *p* < 0.05)
5	( *r* = −0.3) ( *p* < 0.01)	( *r* = −0.3) ( *p* < 0.01)	( *r* = −0.3) ( *p* < 0.01)	( *r* = −0.4) ( *p* < 0.05)	( *r* = −0.4) ( *p* < 0.01)	( *r* = −0.4) ( *p* < 0.05)
6–8	All agonists (NS)			All agonists (NS)		

Abbreviations: ADP, adenosine diphosphate; Annexin V, phosphatidylserine positivity; CRP-XL, collagen-related peptide; NS, not significant; PAC-1, fibrinogen receptor activity; ratio, percentage positive platelets.

It is evident from Table 6 that with an exception (fraction number 3 [both MFI, ratio] α-thrombin), spontaneous annexin V (ratio) of semidense platelets (i.e., fractions numbers 3–5) was associated inversely with WB PAC-1 (both MFI, ratio) agonist reactions in vitro. For fraction number 5, PAC-1 (MFI) (CRP-XL, ADP) and PAC-1 (ratio) (CRP-XL), the significances when comparing the reactivity _high_ and _low_ groups were *p <* 0.01, otherwise they proved to be lower ( *r* range [−0.2 to −0.4]) ( *p <* 0.05). Fraction number 1 platelets behaved differently in that their spontaneous surface annexin V (ratio) related positively with PAC-1 responses after in vitro agonist provocation (α-thrombin PAC-1 [MFI] *r =* 0.2, [ratio] *r =* 0.5, ADP PAC-1 [MFI] *r =* 0.2, PAC-1 [ratio] *r =* 0.4; all *p <* 0.05; Table 6 ).

Table 7 compares surface annexin V (MFI, ratio) of density-separated platelets and WB platelet surface LAMP-1 ([MFI] left, [ratio] right) following agonist provocation. Annexin V (MFI) expressions of platelet density fractions numbers 1 to 6, 8 and 2 to 5, 8 were associated inversely with WB CRP-XL-evoked LAMP-1 (MFI) and LAMP-1 (ratio), respectively ( *r* ranges [−0.2 to −0.4]). For subfractions numbers 4, 5 (annexin V [MFI] vs. LAMP-1 [MFI]) and numbers 2 to 5, 8 (annexin V [MFI] vs. LAMP-1 [ratio]), the levels of significance reached ( *p <* 0.01), else the *p* -values were *p <* 0.05. Annexin V (MFI) expressions of the subfractions linked inversely with WB ADP-induced LAMP-1 (MFI) and LAMP-1 (ratio) as well. For the fractions numbers 1 to 6, 8 ( *r* range [−0.4 to −0.5] (fractions number 1 [ *p <* 0.05] and number 3 [ *p <* 0.001], else the *p-* values were *p <* 0.01). The corresponding figures for LAMP-1 (ratio) proved to be numbers 2 to 6, 8 ( *r* range [−0.4 to −0.5] fraction number 4 [ *p <* 0.01] else [ *p <* 0.05]) ( Table 7 ). Table 7 shows associations between surface annexin V (ratio) of circulating platelet density subfractions and α-thrombin-provoked LAMP-1 (MFI) [fraction number 2] ( *r* = 0.3) and LAMP-1 (ratio) [fractions numbers 4, 5] (both *r* = −0.2) (all *p <* 0.05). Annexin V (ratio) of fractions numbers 3 to 5 linked with WB CRP-XL-evoked LAMP-1 ([ratio] [ *r* range 0 to −0.1]) ( *p <* 0.05) ( Table 7 , right). Finally, inverse relations were established between annexin V (ratio) of fractions (numbers 3–6) and ADP-evoked LAMP-1 (ratio) (all *r* = −0.4) ( *p <* 0.05) ( Table 7 , right).

**Table 7:** ( **A** and **B** ) Summarize how unstimulated surface annexin V in eight density-defined subfractions of normal-sized platelets relate to subsequent whole blood LAMP-1 responses after stimulation with α-thrombin (10 μM), collagen-related peptide (0.15 μg/mL), or adenosine diphosphate (5 μM). In ( **A** ), subfraction annexin V is expressed as mean fluorescence intensity, whereas ( **B** ) presents the ratio of positive platelets. The corresponding whole blood responses are shown as mean fluorescence intensity in the left-hand panels and as ratio in the right-hand panels. The data were analyzed in two separate ways. ( **A** ) Correlation coefficients ( *r* ) were derived by comparing basal in vitro surface annexin V in each platelet subfraction with agonist-triggered whole blood LAMP-1 responses in normal-sized platelets. ( **B** ) The 35 participants were divided according to the magnitude of whole blood LAMP-1 reactivity into a more reactive group ( *n* = 17) and a less reactive group ( *n* = 18). Surface annexin V (both mean fluorescence intensity, ratio) of every subfraction was compared between the LAMP-1 response groups ( *n* = 2) by means of the Student’s *t* -test, and the resulting *p* -values are reported in the tables. These *p* -values arise from the group comparisons alone and are not derived from the correlation analyses.

Density fractions	Mean fluorescence intensity after provocation			Percentage positive cells after provocation		
	α-thrombin	CRP-XL	ADP	α-thrombin	CRP-XL	ADP
	(10 μM)	(0.15 μg/mL)	(5 μM)	(10 μM)	(0.15 μg/mL)	(5 μM)
**A. Annexin V (mean fluorescence intensity) of normal-sized density-separated circulating platelets vs. whole blood agonist-evoked LAMP-1 of normal-sized platelets**						
1	(NS)	( *r* = −0.4) ( *p* < 0.05)	( *r* = −0.4) ( *p* < 0.05)		All agonists (NS)	
2	( *r* = −0.4) ( *p* < 0.05)	( *r* = −0.3) ( *p* < 0.05)	( *r* = −0.5) ( *p* < 0.01)	(NS)	( *r* = −0.3) ( *p* < 0.01)	( *r* = −0.5) ( *p* < 0.05)
3	(NS)	( *r* = −0.3) ( *p* < 0.05)	( *r* = −0.5) ( *p* < 0.001)	(NS)	( *r* = −0.3) ( *p* < 0.01)	( *r* = −0.5) ( *p* < 0.05)
4	(NS)	( *r* = −0.3) ( *p* < 0.01)	( *r* = −0.4) ( *p* < 0.01)	( *r* = −0.40) ( *p* < 0.05)	( *r* = −0.3) ( *p* < 0.01)	( *r* = −0.5) ( *p* < 0.01)
5	(NS)	( *r* = −0.3) ( *p* < 0.01)	( *r* = −0.4) ( *p* < 0.01)	(NS)	( *r* = −0.3) ( *p* < 0.01)	( *r* = −0.5) ( *p* < 0.05)
6	All agonists (NS)	( *r* = −0.2) ( *p* < 0.05)	( *r* = −0.4) ( *p* < 0.01)	(NS)	NS	( *r* = −0.4) ( *p* < 0.05)
7		All agonists (NS)			All agonists (NS)	
8	(NS)	( *r* = −0.2) ( *p* < 0.05)	( *r* = −0.5) ( *p* < 0.01)	(NS)	( *r* = −0.2) ( *p* < 0.01)	( *r* = −0.5) ( *p* < 0.05)
**B. Annexin V (ratio) of normal-sized density-separated circulating platelets vs. whole blood agonist-evoked LAMP-1 of normal-sized platelets**						
1	(NS)	(NS)	( *r* = −0.4) ( *p* < 0.05)	All agonists (NS)		
2	( *r* = −0.4) ( *p* < 0.05)	(NS)	(NS)		All agonists (NS)	
3	(NS)	(NS)	( *r* = −0.4) ( *p* < 0.05)	(NS)	( *r* = −0.1) ( *p* < 0.05)	( *r* = −0.4) ( *p* < 0.05)
4	(NS)	(NS)	( *r* = −0.4) ( *p* < 0.05)	( *r* = −0.2) ( *p* < 0.05)	( *r* = 0) ( *p* < 0.05)	( *r* = −0.4) ( *p* < 0.05)
5	(NS)	(NS)	( *r* = −0.4) ( *p* < 0.01)	( *r* = −0.2) ( *p* < 0.05)	( *r* = 0) ( *p* < 0.05)	( *r* = −0.4) ( *p* < 0.05)
6–8	All agonists (NS)			All agonists (NS)		

Abbreviations: ADP, adenosine diphosphate; Annexin V, phosphatidylserine positivity; CRP-XL, collagen-related peptide; LAMP-1, lysosomal-associated membrane protein; NS, not significant; ratio, percentage positive platelets.

## Discussion

In T2DM surface phosphatidylserine as judged from annexin V (both MFI, ratio) of many circulating platelets unprovoked in vitro, increased agonist-evoked platelet annexin V (MFI) in the test tube ( Table 5 left). The agonists (α-thrombin [10 μM], CRP-XL [0.15 μg/mL], ADP [5 μM]) showed similar behavior. Spontaneous annexin V (MFI, ratio) of most platelets further affected WB agonist-evoked fibrinogen receptor (PAC-1) (MFI, ratio) activation (all agonists) ( Table 6 ) and lysosomal exocytosis (LAMP-1) (MFI, ratio) (CRP-XL, ADP only) ( Table 7 ), contrariwise. Such results do not prove causality but circulating phosphatidylserine-expressing platelets may well affect agonist-evoked platelet activity in vitro (i.e., reactivity).

Visually, after in vitro provocation, surface annexin V (ratio) of normal-sized platelets appeared to have less inverse impact upon fibrinogen receptor responses, i.e., PAC-1 (MFI, ratio) ( Table 6 ). It is, however, partly a chimera as platelet density with respect to counts displays a bell-shaped distribution with higher and lower density fractions containing fewer platelets. 10 16 Thus, the significances when comparing surface annexin V (ratio) of fractions numbers 3–5 and agonist-provoked PAC-1 (MFI, ratio) ( Table 6 ) involved a large quantity of the normal-sized platelet population. Although anucleate, contemporary scientific reports show that platelets experience programmed cell death via apoptosis then demonstrating an enhanced phosphatidylserine expression. 25 26 Procoagulant platelets are characterized by elevated surface phosphatidylserine combined with activation of the P-selectin (CD62P) receptor following dual stimulation with strong agonists. 1 In this study, a physiology activation model was employed using lower agonist concentrations. Due to a technical issue, assessment of the activated P-selectin receptor was not feasible. As a result, throughout the manuscript, if possible, the term “procoagulant platelets” was omitted.

It is tenable that in T2DM either the stimulated receptor or other platelet features affected pathways and magnitudes of agonist-evoked reactions in the test tube (i.e., reactivity). Table 5 (left) backs the latter concept in showing that all agonists displayed close relationships between surface annexin V (MFI) of nearly all circulating platelets, unprovoked in vitro *,* and WB agonists-induced phosphatidylserine (annexin V [MFI]). Further, significant inverse associations between membrane annexin V (MFI) of almost all subfractions and agonist-induced PAC-1 (MFI, ratio) (all used agonists) ( Table 6 ) and to a lesser extent with LAMP-1 (MFI, ratio) (CRP-XL, ADP only) responses ( Table 7 ) indicated that agonist receptors were less important after in vitro provocation.

Spontaneous platelet activity markers of density fraction number 7 were unrelated to WB platelet agonist-induced responses (i.e., reactivity) ( Tables 5 – 7 ). In contrast, the tables show that surface annexin V (MFI) of adjacent density fractions (numbers 6, 8) were linked to reactivity. The consistency made us theorize that fraction number 7 platelets differ with respect to function.

When analyzing platelet activity makers with a flow cytometer, it is possible to present data either as MFI or as the proportion (%) of fluorescence positive corpuscles (ratio). It is anticipated that the approaches yield equivalent results. Indeed, congruence existed when determining the consequences of platelet subpopulation annexin V (MFI, ratio) upon agonist-evoked PAC-1 (MFI, ratio) and LAMP-1 (MFI) (only CRP-XL, ADP) activities ( Tables 5 and 6 ). In contrast, diverging outcomes when comparing subfraction annexin V (MFI, ratio) and phosphatidylserine-positive platelets after provocations (all agonists) ( Table 5 left and right) indicate that annexin V [MFI, ratio] in this context reflected different platelet features. In samples with lower and higher platelet counts, the flow cytometer collected platelets differently (see Materials and Methods). Thus, we used the ratio (%) of positive platelets after provocation as an experimental parameter, but its replacement with the absolute counts of fluorescence positive normal-sized platelets could be more accurate.

To judge the implications of the current basic science for T2DM is difficult. It defines possible new regulatory mechanisms for WB platelet reactivity in vitro and it may well be important. If platelets behave similarly in vivo it could be an option to suppress circulating phosphatidylserine-expressing platelets. It can then be hypothesized that such an inhibition could augment the activity of fibrinogen receptors and lysosomal exocytosis of circulating platelets ( Tables 6 and 7 ).

In T2DM, surface annexin V (MFI) of almost all normal-sized circulating platelets affected agonist-evoked fibrinogen (PAC-1) (MFI, ratio) receptor activity, contrariwise ( Table 6 ). In addition, Table 6 shows that surface annexin V (ratio) of the fractions (numbers 3–5), i.e., many circulating platelets performed alike in associating inversely with WB agonist-provoked PAC-1 (MFI, ratio). Further, surface annexin V (MFI) of fractions (numbers 3–5) affected LAMP-1 (ratio) after WB CRP-XL and ADP in vitro provocations ( Table 7 ). It made us conclude that exposed phosphatidylserine (i.e., annexin V [MFI]) of most platelets influenced agonist-induced surface PAC-1 and LAMP-1 ex vivo, contrariwise.

In summary, in patients with T2DM, circulating normal-sized phosphatidylserine-expressing platelets—as determined by surface annexin V (MFI)—exhibited enhanced WB agonist-induced surface phosphatidylserine exposure in vitro. Following stimulation, these platelets also exerted an inverse effect on both fibrinogen receptor activity and lysosomal exocytosis. The agonists tested (α-thrombin [with certain exceptions], CRP-XL, and ADP) produced comparable outcomes. These observations constitute novel findings, but they do not establish causality. In this setting, however, surface phosphatidylserine of circulating normal-sized platelets, unprovoked in vitro, was found to affect WB T2DM platelet reactivity in the test tube.

## Limitations

The present laboratory investigation, which included a considerable cohort of individuals with T2DM ( *n* = 35), is subject to certain limitations. We employed Ca ^2+^ -sensitive annexin V to identify surface phosphatidylserine, and all samples were recalcified prior to analysis. However, before and during platelet gradient preparations, the subfractions were exposed to EDTA and platelet inhibitors. 10 11 12 Such agents could make the recalcification of the subpopulations less effective, potentially diminishing surface expression of annexin V. In this case, lactadherin could represent a more appropriate membrane-exposed phosphatidylserine marker.

Clinical characteristics among routine patients varied ( Table 1 ), but the sample size ( *n* = 35) was insufficient to allow for confounder adjustment. The handling of platelet inhibitors—asp *i* rin ( *n* = 8) and clopidogrel ( *n* = 1)—remains open to debate, but to accurately reflect primary care T2DM patients, no exclusion criteria were applied. Aspirin did neither influence the expression of annexin V of circulating, normal-sized platelets nor agonist evoked platelet reactions in WB (data not shown); consequently, such prescriptions were not regarded as exclusion criteria. While the increased diabetic cohort enhanced statistical power, funding constraints precluded the inclusion of healthy controls. Thus, this study pertains exclusively to T2DM patients, and its findings cannot be extrapolated to nondiabetic seniors.

## Conclusions

In T2DM, circulating normal-sized phosphatidylserine-expressing platelets none-provoked in vitro influenced WB agonist-evoked reactions in vitro, i.e., reactivity. After agonist provocation such platelets favored platelet phosphatidylserine expression, downregulation of fibrinogen receptors, and decreased lysosomal exocytosis. It is concluded that in T2DM, phosphatidylserine activity of circulating platelets associates with WB platelet reactivity.

**What is known about the topic?**

- Little is known about the regulation of platelet responses after in vitro agonist provocation in type 2 diabetes.
